# Supplementary material for: Leukocytospermia induces intraepithelial recruitment of dendritic cells and increases SIV replication in colorectal tissue explants
Source: Commun Biol. 2021 Jul 12;4:861. doi: 10.1038/s42003-021-02383-9 (PMC8275775; doi:10.1038/s42003-021-02383-9)
Supplement: Supplementary file 2 — Supplementary Informations [file 42003_2021_2383_MOESM2_ESM.pdf]

Supplementary Information for

Leukocytospermia induces intraepithelial recruitment of dendritic cells and increases SIV replication in colorectal tissue explants

Authors: Mariangela Cavarelli, Stéphane Hua, Naima Hantour, Sabine Tricot, Nicolas Tchitchek, Céline Gommet, Hakim Hocini, Catherine Chapon, Nathalie Dereuddre-Bosquet, and Roger Le Grand

Corresponding author: Mariangela Cavarelli, CEA, IDMIT Department, 18 route du Panorama 92265, Fontenay-aux-Roses, France.
Phone: +33146548027
E-mail: mariangela.cavarelli@cea.fr

This pdf includes:

1. Supplemental Results

Tissue explant model optimization

2. Supplemental Figures 1 to 7

3. Supplemental Tables 1 and 2

Supplemental Results

Tissue explant model optimization

Explants were individually exposed to the various stimuli for 2 h in a polarized manner and then washed, transferred to a collagen sponge Gelfoam$^{(R)}$ raft, and incubated for up to 12 days (**Supplementary Figure 3A**). The SIVmac251 virus stock was titrated to determine the infectious dose required to infect 100% of the explants while avoiding excessive viral replication and cytotoxicity (**Supplementary figure 4A**). Infection of the explants was further confirmed by coculturing uninfected indicator cells (CEMx174) with the cells emigrating from the colonic explants 24 h after culture (**Supplementary figure 4B**). After removal of the migratory cells, the remaining explants were cultured for another 11 days before measuring SIV replication. Based on the results, 5 x 10^6 copies/ml of SIVmac251 for used for further experiments. Athough the tissue remained viable for 12 days in culture, the structural architecture of the colonic explants degenerated over time under all conditions, including control treatment with medium (**Supplementary figure 3B, C**), in accordance with the results of others [1,2], indicating that it was associated with general deterioration of the tissue rather than a specific treatment. In addition, post hoc analysis of uncultured tissues (explant fixed at the baseline of the experiment) was performed to evaluate the intactness of the epithelium. Experiments showing damage to the epithelium at baseline were excluded from the analysis.

Supplemental Figures

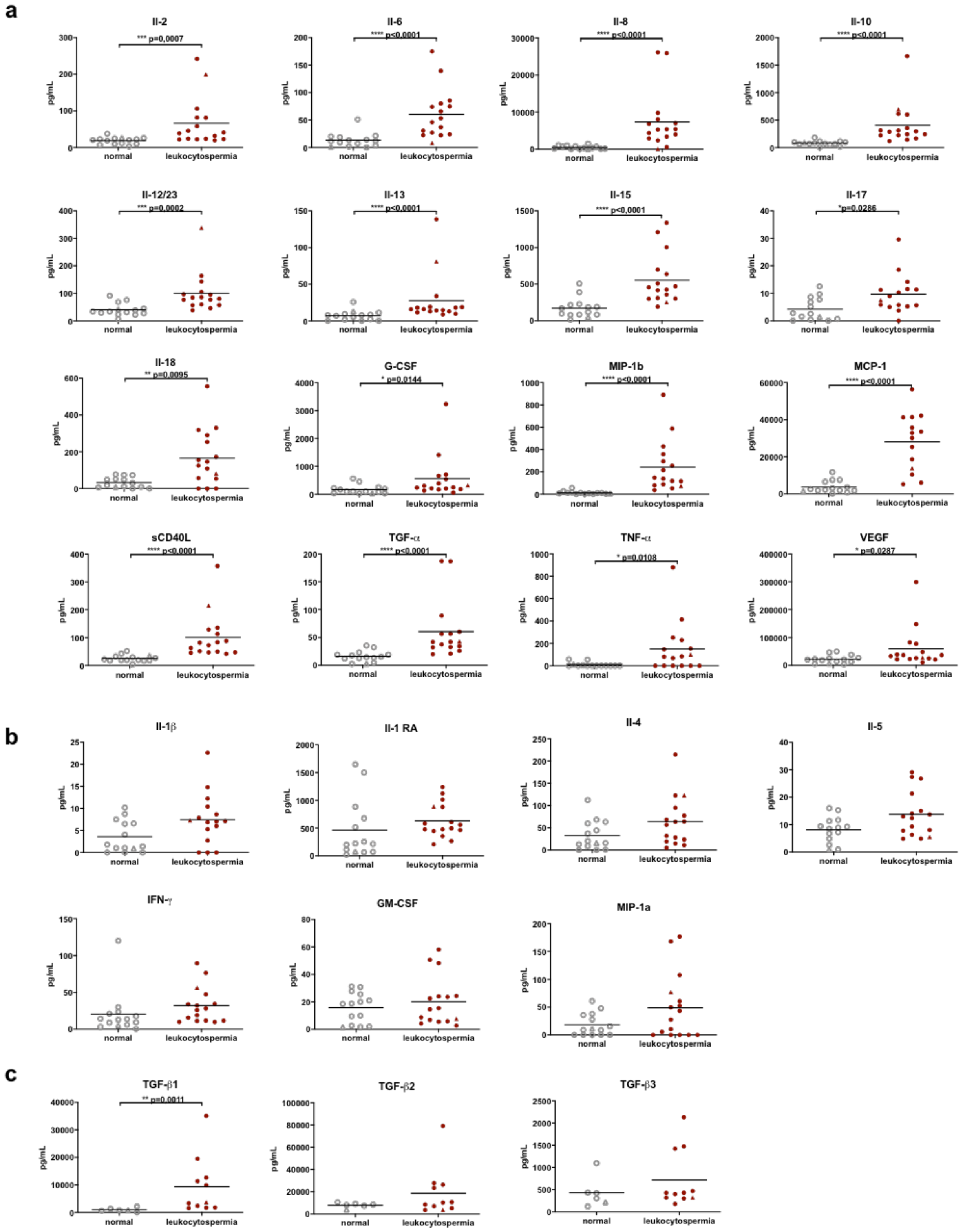

Supplementary Figure 1. Cytokines and chemokines affected, or not, by leukocytospermia of the seminal plasma of SIV- macaques.

Mean and SEM of the level of 23 cytokines and chemokines in seminal plasma of 13 normal (NS, grey symbols) and 15 leukocytospermic (LS, red symbols) uninfected macaques (**A-B**) and the level of TGF-ß 1-2-3 in five NS and 10 LS animals (**C**). Triangles indicate the values of the seminal plasma pool. Statistical significance between conditions was tested using Wilcoxon signed rank tests, *p < 0.05, **p < 0.01, ***p < 0.001, and ***p < 0.0001.

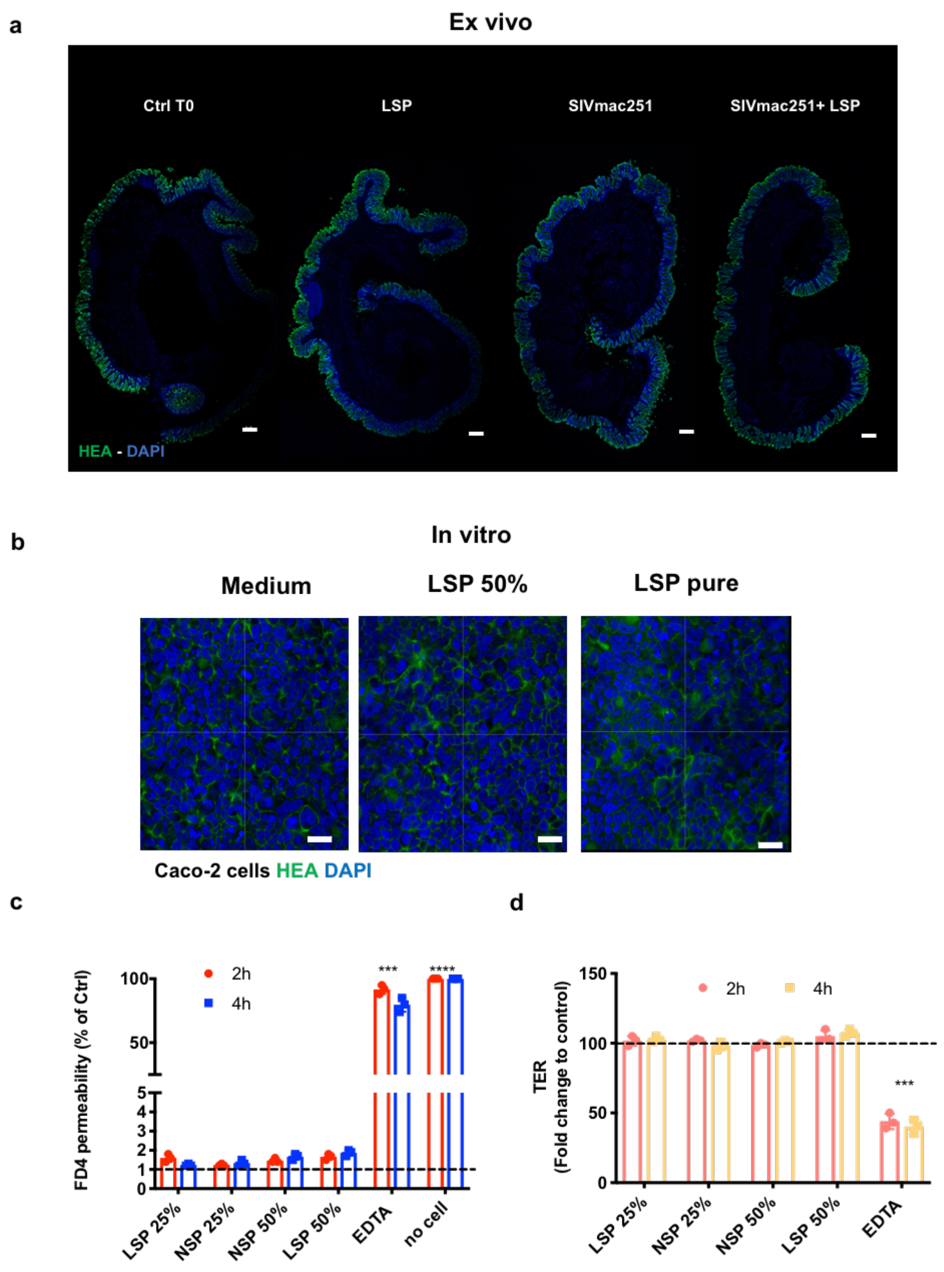

Supplementary Figure 2. ***Ex vivo*** **and** ***in vitro*** **evaluation of seminal plasma toxicity and its impact on epithelial permeability.**

A) Immunofluorescence staining for the human epithelial antigen (HEA-FITC, green) of explants before and after 2 h treatment with leukocytospermic seminal plasma (LSP, 25% dilution) or SIVmac251, with or without 25% LSP, compared to that of control (ctrl) explants (fixed at baseline). Nuclei are stained with DAPI. The entire explant is shown. Scale bar 200 µm.

B) Immunofluorescence staining of a tight monolayer of intestinal epithelial cells (Caco-2 cells) after 4 h of culture with or without 25% and 50% LSP. Human epithelial antigen (HEA) staining is shown (green). Nuclei are stained with DAPI. Magnification = 40x. Scale bar = 25 µm. **C)**

Integrity of the epithelial barrier measured by the addition of Dextran-FITC (FD4, 4kDa, 250 µg/ml) to the apical side of the Caco-2 monolayer and incubation for 2 or 4 h. Results are shown as the percentage of the positive control (i.e. FD4 added to the upper chamber of the transwell without Caco-2 cells). **D**) Change in transepithelial resistance (TER) after 2 or 4 h of exposure to 25% or 50% NSP or LSP. In C and D, 100 mM EDTA was used as a positive control for junction disruption. Results are shown as the percentage of control TER (medium-treated cells). Statistical significance between conditions was tested using Wilcoxon signed rank tests, ***p 0.001, ****p < 0.0001. Results are presented as the mean values ± SD of triplicates from a representative experiment of three.

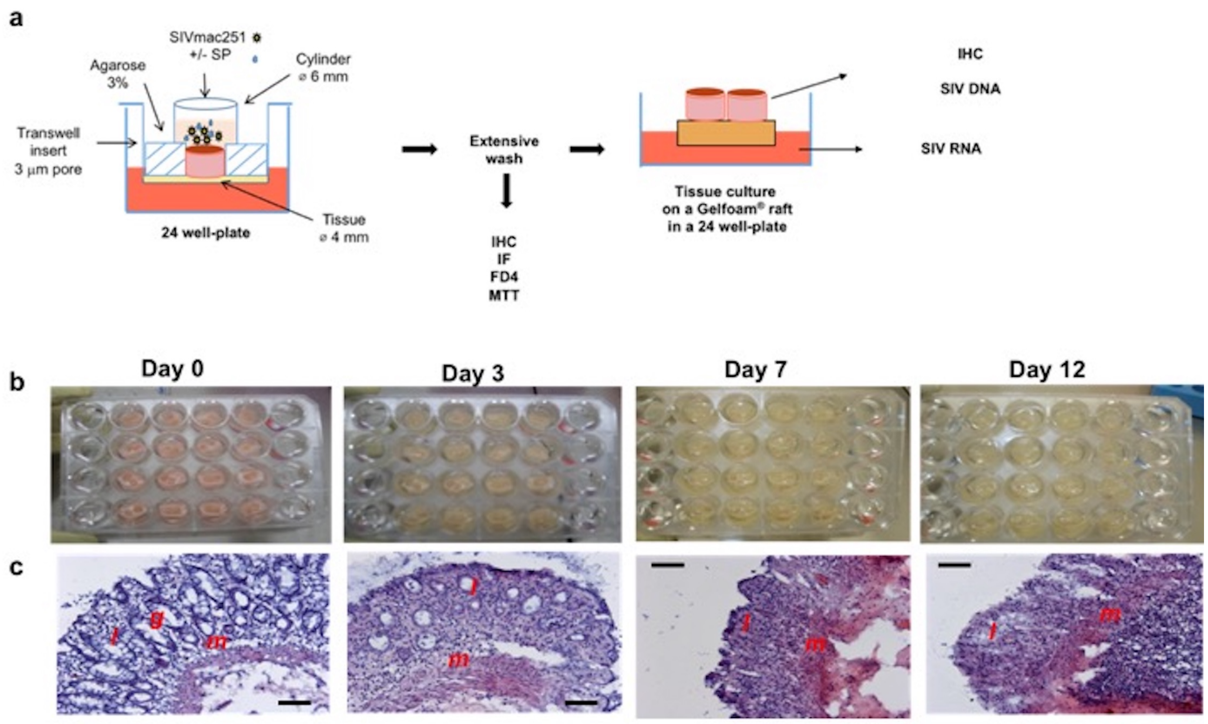

Supplementary Figure 3. Schematic representation of the experimental system used for the *ex vivo* infection and culture of polarized sigmoid colonic tissue.

A) Colonic tissue fragments were cut with a biopsy punch into circular pieces (4 mm in diameter), including the epithelium and submucosa, and placed with the epithelial luminal side up on top of a permeable membrane in a two-chamber system. The edges around the explant were sealed with 3% agarose and a polyester cylinder (6 mm diameter) was inserted into the agarose, resulting in the creation of a sealed and polarized apical chamber. Explants were exposed to SIVmac251 viral isolates with or without seminal plasma. At the end of the incubation, the chambers were extensively washed to remove the virus and the seminal plasma and explants were either subjected to FD4 and MTT assay, or immediately fixed to be analyzed by immunohistochemistry (IHC) or immunofluorescence (IF), or transferred onto a medium-saturated Gelfoam(R) sponge (2 donor matched explants/sponge) in a 24 well-plate and maintained in culture for up to 12 days. Explants were subjected to IHC and the pro-viral load was determined at 12 dpi. Culture supernatants were used to determine the SIV viral load. **B**) Image of a 12 well-plate containing explants at days 0, 3, 7, and 12 of culture. **C**) Medium-treated explants at days 0, 3, 7, and 12 post-culture were fixed in 4% PFA, cryopreserved, sectioned, and stained with Hematoxylin-Eosin to evaluate general tissue architecture, cell

integrity, and necrosis over the culture period. Colonic glands (*g*), the lamina propria (*l*) and the muscolaris mucosa (*m*) are indicated. Scale bar = 50 μm.

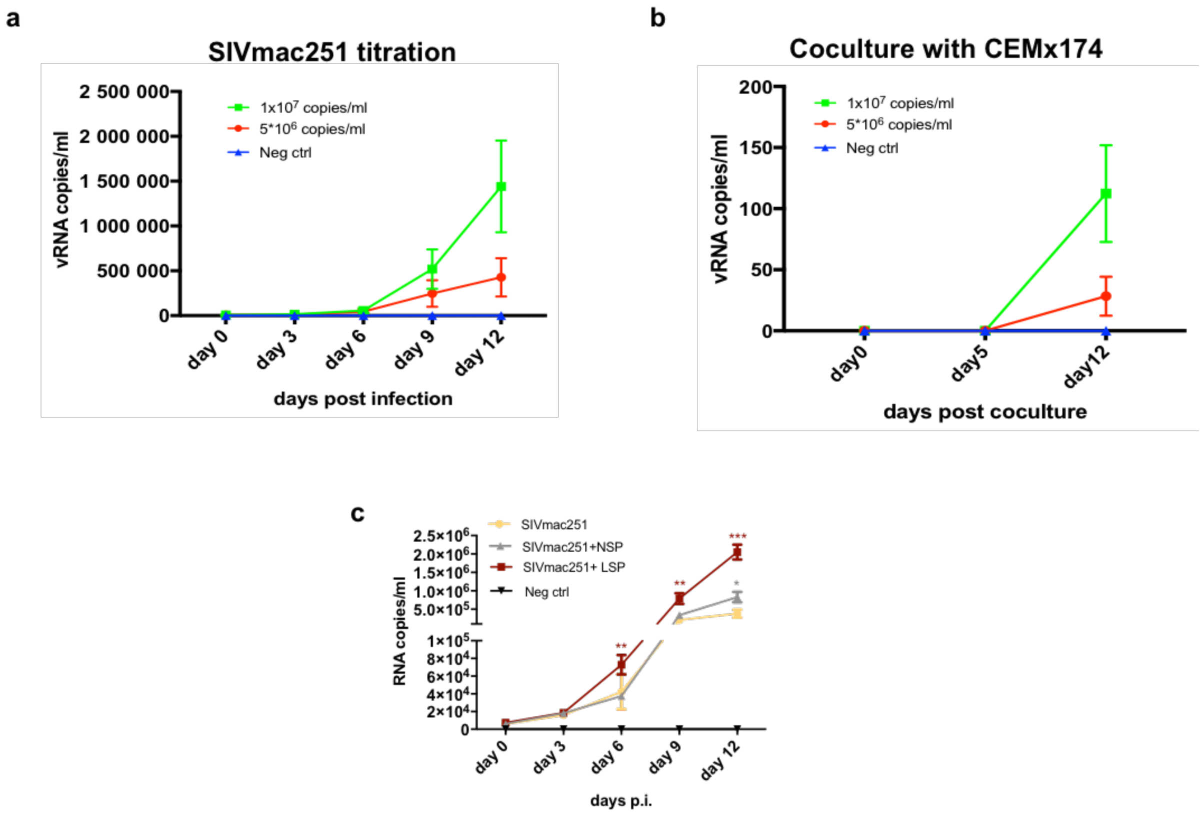

Supplementary Figure 4. Set-up of the *ex vivo* sigmoid colon infection and the influence of seminal plasma on SIVmac251 replication.

A) Explants were incubated for 2 h with various concentration of SIVmac251 in a polarized manner and then washed and cultured on Gelfoam$^{(R)}$ sponges. Culture supernatant was collected at days 0, 3, 7, and 12 and viral replication assessed by qRT-PCR. Data represent the mean and SEM of triplicates from three independent experiments. **B**) Cells that migrated out from the tissue were collected 24 h post-culture and cocultured with uninfected CEMx174 cells at a 1:1 ratio to determine the transfer of infection. Viral load was measured by qRT-PCR over 12 days of coculture. Data represent the mean and SEM of triplicates from three independent experiments. **C**) Kinetics of SIVmac251 replication in explants treated with virus alone (yellow line), virus plus 25% of an individual LSP (red line), or virus plus 25% of an individual NSP (gray line). Culture medium was used as a negative control. Virus replication was evaluated in the basal supernatant as viral RNA copies/ml. Infection was carried out using NSP or LSP from one animal to infect tissues from 3 different donors. Results are presented as the mean values ± SEM of triplicates from three independent experiments. Statistical significance between SIVmac251 plus seminal plasma and SIVmac251 alone was tested using Friedman tests with post-hoc Benjamini, Krieger, and Yekutieli tests.

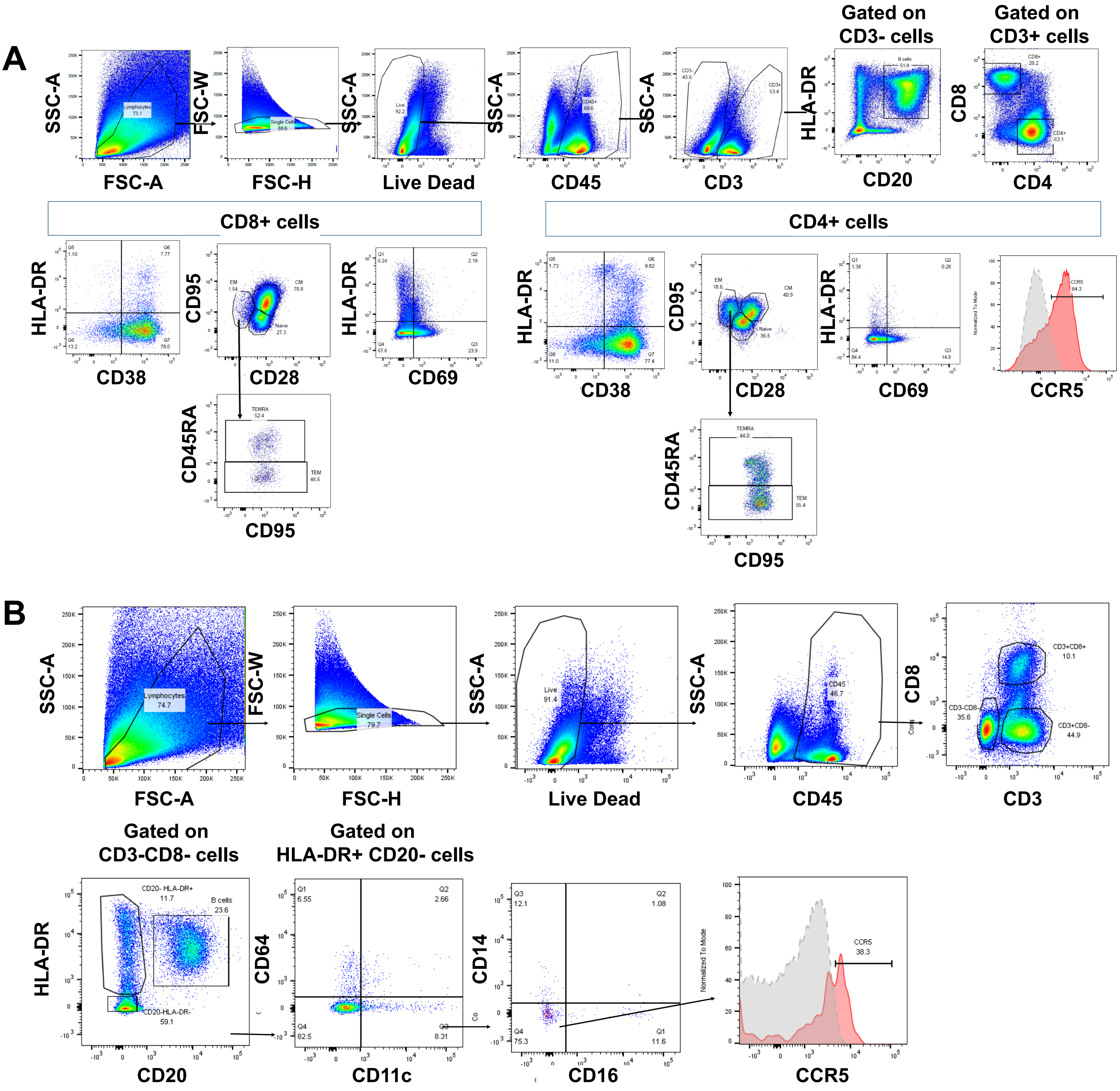

Supplementary Figure 5. Gating strategy used to identify lymphocytes and dendritic cells among colonic lamina propria mononuclear cells.

Representative dot plots from an individual healthy cynomolgus macaque are shown. **A)** Live leukocytes were first identified based on FSC and SSC characteristics, singlets, live gating and CD45 expression. CD4 and CD8 T cells were identified by CD3 expression and CD4 or CD8 expression. In each subpopulation, the activation state was characterized based on the expression of CD38, HLA-DR and CD69, and the memory phenotype was assessed by the expression of CD28, CD95 and CD45RA (Naive: CD28+CD95-, Central memory: CD28+CD95+, Effector memory: CD28-CD95+CD45RA-, TEMRA: CD28-CD95+CD45RA+). Furthermore, CCR5 expression was monitored on CD4 T cells (FMO is represented as a gray layout and sample as red layout). **B)** From live leukocytes, antigen presenting cells were identified based on lacking the expression of CD3, CD8 and CD20 and expressing HLA-DR. Myeloid DCs were then identified as lacking CD64, CD14 and CD16 expression and expressing CD11c. CCR5 expression was monitored on this subset (FMO is represented as a gray layout and sample as red layout).

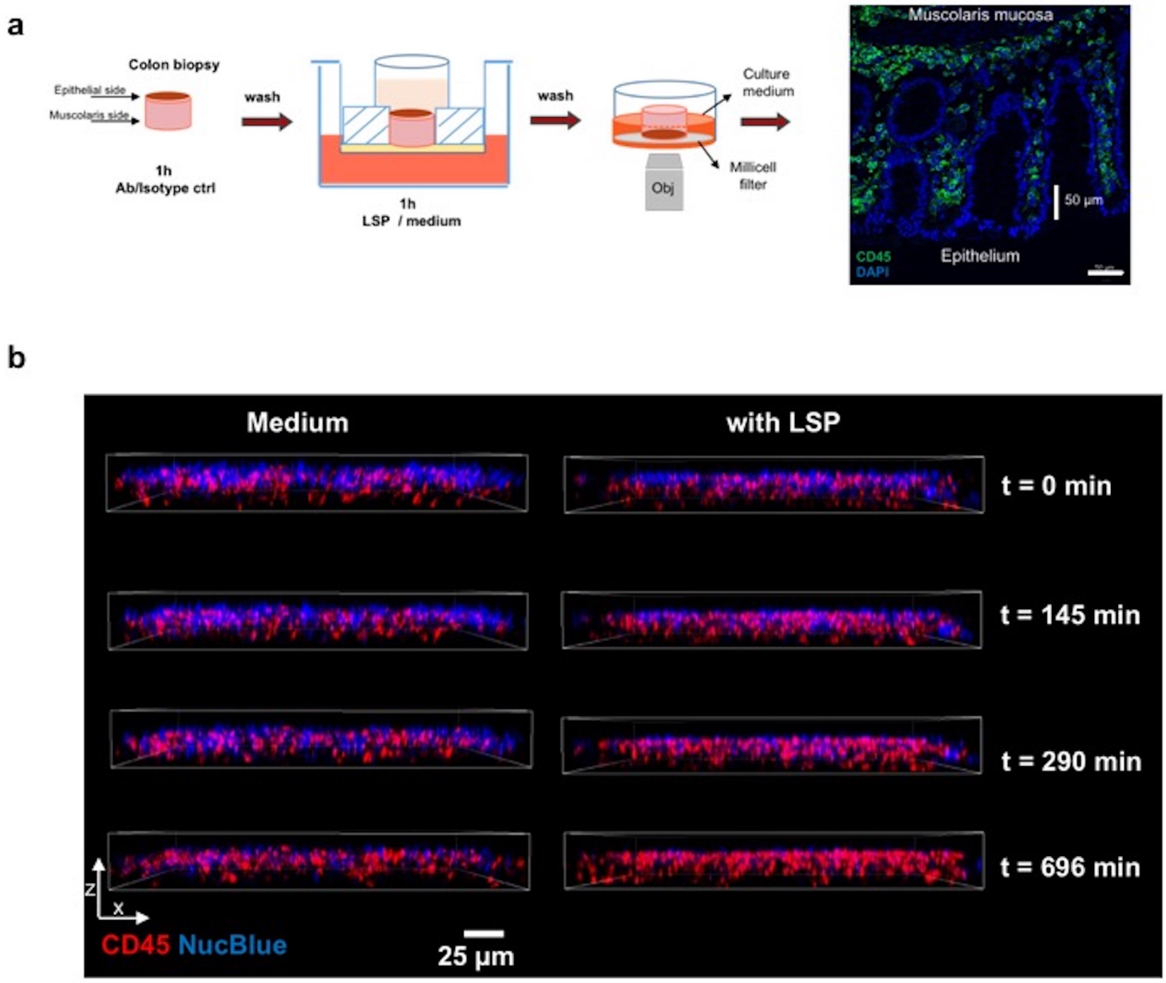

Supplementary Figure 6. Time-lapse confocal microscopy and LSP-induced leukocyte recruitment.

A) Schematic representation of the experimental procedure used to perform time-lapse confocal laser scanner microscopy of tissues treated, or not, with LSP. A confocal microscopy image of the colonic mucosa labelled with mouse anti-human CD45 antibody shows the steady-state distribution of leukocytes inside the colonic lamina propria. Nuclei are stained with DAPI. The scale bar of 50 µm indicates the size of the z-stack acquired by time-lapse confocal fast-laser scanning microscopy. **B**) Three-dimensional rendering of representative fields obtained using NIS-Elements AR Analysis 5.02.0 (NIKON) and Image J software. The mouse anti-human CD45 antibody shows the leukocytes (red) and NucBlue dye label the nuclei (blue). Explants were either exposed to complete medium or medium plus 25% LSP for 1h, then submitted for video-microscopy. Representative images obtained at the beginning of the experiment, after 145, 290, and 696 minutes of acquisition are shown.

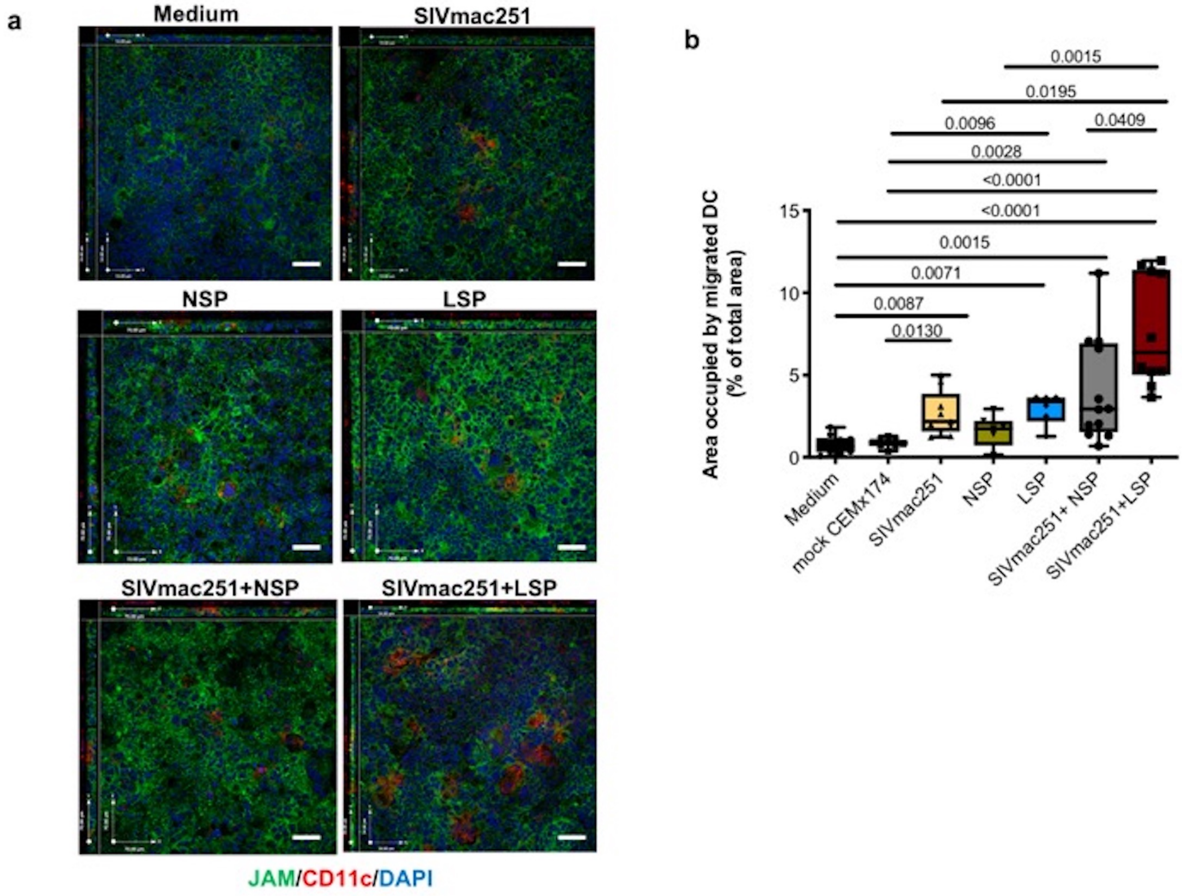

Supplementary Figure 7. Dendritic cell migration across a Caco-2 cells monolayer. Caco-2/DCs were incubated with SIVmac251, 25% NSP, 25% LSP, SIVmac251+25% NSP, or SIVmac251+25% LSP. Medium (DMEM 10% FCS) and supernatants from uninfected CEMx174 cells were used as negative controls. **A**) Confocal microscopy cross-sectional images of specimens stained for DCs (CD11c, red) and the epithelial junction adhesion molecule (JAM, green). Nuclei are stained with DAPI. Results are from one representative experiment of three. **B**) Quantitative analysis of DC migration across the Caco-2 cell monolayer. Results are expressed as the percentage of the area occupied by DCs relative to that of the whole field. Bars represent the mean ± SD of three or four fields from two or three different experiments. Statistical significance between the different conditions was tested using Kruskal-Wallis tests with post-hoc Benjamini, Krieger, and Yekutieli tests. Scale bar = 50 μm.

Supplemental Tables

Supplementary Table 1. List of antibodies for immunofluorescence and confocal microscopy of sigmoidal tissues, Caco-2 cells, and DCs.

Cell type	Antibody	Clone	Supplier
Antigen presenting cells	HLA-DPDQDR	CR3/43	Dako
Leukocytes	CD45	HI30	BD pharmingen
Tight junctions	Junction Adhesion Molecule (JAM) A	BV16	Hycult Biotech
Adherent junctions	E-Cadherin	36/E-Cadherin	BD Trans. Lab.
Epithelial cells	Anti-epithelial antigen (HEA)	Ber-EP4	Dako
DCs	CD11c	BU15	Immunotech

Supplementary Table 2. List of antibodies used to characterize colonic lamina propria cells by flow cytometry

Antibody	Fluorochrome	Clone	Supplier
CD14	Alexa-700	M5E2	BD pharmingen
CD69	Alexa-700	FN50	BD pharmingen
HLA-DR	APC-H7	G46-6	BD pharmingen
CD16	BUV395	3G8	BD Biosciences
CD3	BUV395	SP34-2	BD Biosciences
CD11c	BV421	3,9	Biolegend
CD95	BV510	DX2	Biolegend
CD20	BV650	2H7	BD Biosciences
CD3	BV650	SP34-2	BD Biosciences
CD8	BV650	RPA-T8	BD Horizon
CCR5	BV786	3A9	BD Biosciences
CD38	FITC	AT-1	Stemcell
CD123	PC7	7G3	BD pharmingen
CD45RA	PC7	L48	BD Biosciences
CD45	PerCp	D058-1283	BD pharmingen
CD28	PE-TexasRed	CD28,2	Beckman Coulter
CD4	V450	L200	BD Horizon
CD64	VioGreen	REA978	Miltenyi Biotech
Blue Viability Dye			Invitrogen

Supplementary References

1. Fletcher, P. S. *et al.* Ex vivo culture of human colorectal tissue for the evaluation of candidate microbicides. *AIDS* **20**, 1237–1245 (2006).
2. Abner, S. R. *et al.* A Human Colorectal Explant Culture to Evaluate Topical Microbicides for the Prevention of HIV Infection. *J. Infect. Dis.* **192**, 1545–1556 (2005).
