# Supplementary material for: Leukocytospermia induces intraepithelial recruitment of dendritic cells and increases SIV replication in colorectal tissue explants
Source: Commun Biol. 2021 Jul 12;4:861. doi: 10.1038/s42003-021-02383-9 (PMC8275775; doi:10.1038/s42003-021-02383-9)
Supplement: Supplementary file 3 — Description of Additional Supplementary Files [file 42003_2021_2383_MOESM3_ESM.pdf]

Description of Additional Supplementary Files

File Name: Supplementary Data 1
Description: The source data underlying Figures 1, 2, 3, 4, 5, 6 and 7.
