# Supplementary material for: Leukocytospermia induces intraepithelial recruitment of dendritic cells and increases SIV replication in colorectal tissue explants
Source: Commun Biol. 2021 Jul 12;4:861. doi: 10.1038/s42003-021-02383-9 (PMC8275775; doi:10.1038/s42003-021-02383-9)
Supplement: Supplementary file 5 — Reporting Summary [file 42003_2021_2383_MOESM5_ESM.pdf]

nature research

Corresponding author(s): Mariangela Cavarelli, COMMSBIO-20-3455-T

Last updated by author(s): Jun 7, 2021

Reporting Summary

Nature Research wishes to improve the reproducibility of the work that we publish. This form provides structure for consistency and transparency in reporting. For further information on Nature Research policies, see our Editorial Policies and the Editorial Policy Checklist.

Statistics

For all statistical analyses, confirm that the following items are present in the figure legend, table legend, main text, or Methods section.

n/a	Confirmed	
☐	☒	The exact sample size (*n*) for each experimental group/condition, given as a discrete number and unit of measurement
☐	☒	A statement on whether measurements were taken from distinct samples or whether the same sample was measured repeatedly
☐	☒	The statistical test(s) used AND whether they are one- or two-sided<br>*Only common tests should be described solely by name; describe more complex techniques in the Methods section.*
☒	☐	A description of all covariates tested
☐	☒	A description of any assumptions or corrections, such as tests of normality and adjustment for multiple comparisons
☐	☒	A full description of the statistical parameters including central tendency (e.g. means) or other basic estimates (e.g. regression coefficient) AND variation (e.g. standard deviation) or associated estimates of uncertainty (e.g. confidence intervals)
☐	☒	For null hypothesis testing, the test statistic (e.g. F, t, r) with confidence intervals, effect sizes, degrees of freedom and P value noted<br>*Give P values as exact values whenever suitable.*
☒	☐	For Bayesian analysis, information on the choice of priors and Markov chain Monte Carlo settings
☒	☐	For hierarchical and complex designs, identification of the appropriate level for tests and full reporting of outcomes
☒	☐	Estimates of effect sizes (e.g. Cohen's d, Pearson's r), indicating how they were calculated

Our web collection on statistics for biologists contains articles on many of the points above.

Software and code

Policy information about availability of computer code

Data collection	flow cytometry: BD DIVA
Data analysis	Data was analyzed using GraphPad Prism v8.0.2 and ImageJ.

For manuscripts utilizing custom algorithms or software that are central to the research but not yet described in published literature, software must be made available to editors and reviewers. We strongly encourage code deposition in a community repository (e.g. GitHub). See the Nature Research guidelines for submitting code & software for further information.

Data

Policy information about availability of data

All manuscripts must include a data availability statement. This statement should provide the following information, where applicable:

- Accession codes, unique identifiers, or web links for publicly available datasets
- A list of figures that have associated raw data
- A description of any restrictions on data availability

The authors declare that all the data supporting the findings of this study are available within the paper and its supplementary information files.

Field-specific reporting

Life sciences study design

All studies must disclose on these points even when the disclosure is negative.

Sample size	No sample size calculation was performed. For all key experiments at least three donors were analysed
Data exclusions	No data has been excluded from analysis
Replication	All findings were reliably reproducible
Randomization	No randomization was necessary
Blinding	No blinding was necessary, however analysis of HE tissue staining and videomicroscopy data was blinded.

Reporting for specific materials, systems and methods

We require information from authors about some types of materials, experimental systems and methods used in many studies. Here, indicate whether each material, system or method listed is relevant to your study. If you are not sure if a list item applies to your research, read the appropriate section before selecting a response.

Materials & experimental systems

n/a	Involved in the study	
☐	☒	Antibodies
☐	☒	Eukaryotic cell lines
☒	☐	Palaeontology and archaeology
☐	☒	Animals and other organisms
☒	☐	Human research participants
☒	☐	Clinical data
☒	☐	Dual use research of concern

Methods

n/a	Involved in the study	
☒	☐	ChIP-seq
☐	☒	Flow cytometry
☒	☐	MRI-based neuroimaging

Antibodies

Antibodies used	All antibodies (including clone number) are described in the methods and Supplementary Informations
Validation	All antibodies were comercially available, specificity had been described by the manufacturer. For human antibodies cross-reactivity with cynomolgus macaques is available from the "Non-Human Primate Reagent Resource" (www.nhpreagents.org)

Eukaryotic cell lines

Policy information about cell lines

Cell line source(s)	Caco-2 cells were kindly provided by Dr. Maria Rescigno, Humanitas Institute, Milan, Italy
Authentication	This cell line has not been authenticated in-house
Mycoplasma contamination	Confirmed negative test for mycoplasma contamination
Commonly misidentified lines (See ICLAC register)	No commonly misidentified cell lines were used in this study

Animals and other organisms

Policy information about studies involving animals; ARRIVE guidelines recommended for reporting animal research

Laboratory animals	This study included 28 cynomolgus macaques (Macaca Fascicularis) for seminal plasma collection and 9 cynomolgus macaques for ex vivo, flow cytometry and confocal microscopy experiments
Wild animals	No wild animals were used in the study. All animals used were purposed bread and imported from AAALAC certified breeding centers
Field-collected samples	No data was collected from the field
Ethics oversight	The study was approved by the local ethics committee (Comité d'Ethique en Experimentation Animale de la Direction des Sciences du Vivant au CEA under numbers: 2015032511332650(APAFIS#373); 10-062; 12-103). Tissue was collected at necropsy.

Note that full information on the approval of the study protocol must also be provided in the manuscript.

Flow Cytometry

Plots

Confirm that:

- [x] The axis labels state the marker and fluorochrome used (e.g. CD4-FITC).
- [x] The axis scales are clearly visible. Include numbers along axes only for bottom left plot of group (a 'group' is an analysis of identical markers).
- [x] All plots are contour plots with outliers or pseudocolor plots.
- [x] A numerical value for number of cells or percentage (with statistics) is provided.

Methodology

Sample preparation	Thoroughly described in manuscipt
Instrument	Acquisition was performed on a BD LSRII flow cytometer
Software	BD FACSDiva softawe and FlowJo 9.8.3 was used for acquisition and analysis
Cell population abundance	Thoroughly described in manuscipt
Gating strategy	Thoroughly described in manuscipt

- [x] Tick this box to confirm that a figure exemplifying the gating strategy is provided in the Supplementary Information.
